# Supplementary material for: Anisotropic organization of circumferential actomyosin characterizes hematopoietic stem cells emergence in the zebrafish
Source: eLife. 2018 Aug 22;7:e37355. doi: 10.7554/eLife.37355 (PMC6105311; doi:10.7554/eLife.37355)
Supplement: Figure 4—source data 1. [file elife-37355-fig4-data1.pdf]

Source Data

EHT				
		Lengh	Width	Area
Map2	-	23,36	20,16	277,709
Map3	-	13,94	14,28	113,75
Map3	-	17	12,92	143,113
Map4	-	15,98	18,02	207,849
Map5	-	20,149	14,624	201,829
Map5	-	11,049	18,849	146,382
Map5	-	13,974	17,874	174,475
Map6	Figure 4	9,99	18,9	114,016

Potentially Hemogenic				
		Lengh	Width	Area
Map1	Figure 3	35,2	14,08	326,451
Map1	Figure 3	53,44	19,52	509,338
Map2	-	67,2	22,4	718,95
Map3	-	80,24	28,9	1109,65
Map3	-	57,12	34	1058,09
Map6	Figure 4	54,27	24,03	754,952
Map6	Figure 4	54	19,44	533,191

Map1	Sequence
Map2	Sequence
Map3	Sequence
Map4	Single time point
Map5	Sequence
Map6	Sequence

Endothelial				
		Lengh	Width	Area
Map1	Figure 3	48,64	38,72	1271,296
Map1	Figure 3	49,28	32,96	1044,173
Map1	Figure 3	59,52	50,24	1457,152
Map1	Figure 3	66,88	26,88	845,21
Map1	Figure 3	41,28	25,92	766,362
Map1	Figure 3	44,8	30,4	605,491
Map1	Figure 3	46,4	26,24	536,576
Map3	-	62,56	33,32	835,326
Map3	-	58,14	40,46	1145,018
Map3	-	54,4	47,6	1244,55
Map3	-	63,58	46,58	1467,542
Map3	-	71,74	36,72	1574,126
Map3	-	53,38	39,1	987,687
Map4	-	62,22	52,36	2118,602
Map4	-	51,68	57,46	1568,924
Map4	-	37,06	55,08	1196,114
Map4	-	93,5	77,18	2096,175
Map5	-	62,072	49,398	1267,902
Map5	-	43,873	35,423	974,294
Map6	Figure 4	40,77	43,74	968,112
Map6	Figure 4	54,54	54	1207,224
Map6	Figure 4	65,61	56,16	1556,123
Map6	Figure 4	56,43	57,24	1551,239
Map6	Figure 4	51,3	67,5	1823,229
Map6	Figure 4	79,65	31,86	1092,844
Map6	Figure 4	59,4	25,11	776,677

Figure 4-source data 1

Figure 4D: Numerical data (width, length and area) and corresponding 2D-Maps with the contours of EHT cells (red), hemogenic cells (blue) and endothelial cells (green).

Map1

A

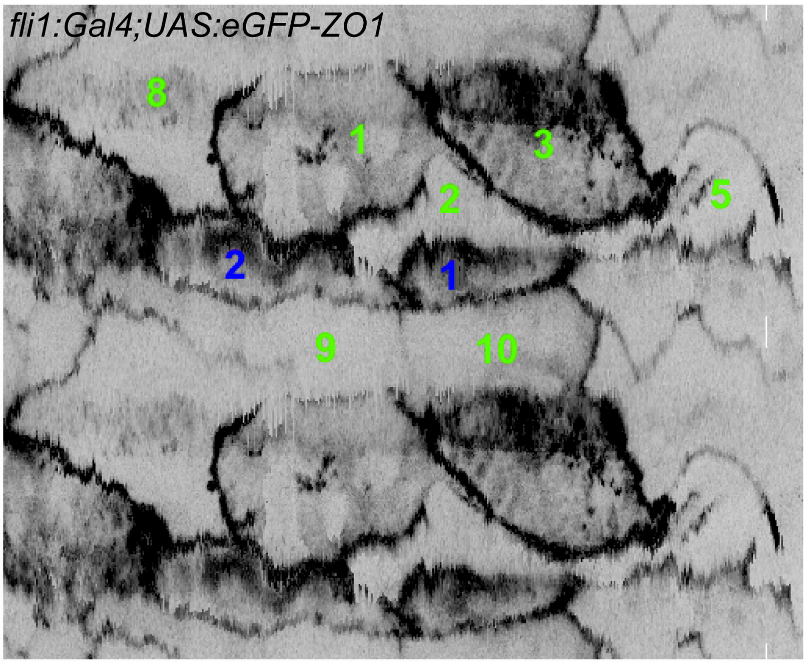

B

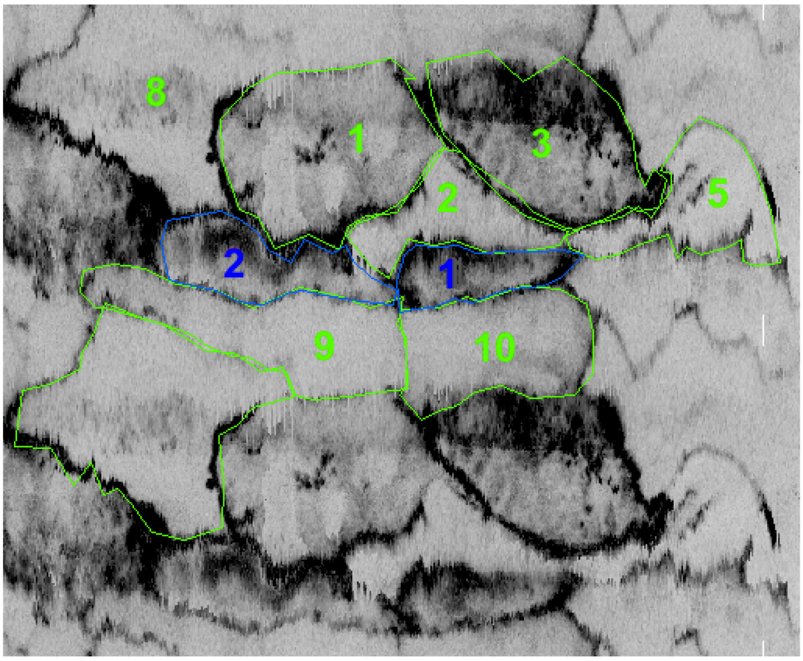

2D projection of DA portion extracted from a confocal TL sequence performed on a 48 hpf *Tg(fli1:Gal4;UAS:RFP; UAS:eGFP-ZO1)* embryo (related to Figure 4-D). (A) Cells numbered in blue and green correspond to “Potentially hemogenic” and “Endothelial” cells respectively. (B) Same image with the “Potentially hemogenic” and “Endothelial” cells contours delimited by hand.

Map 2

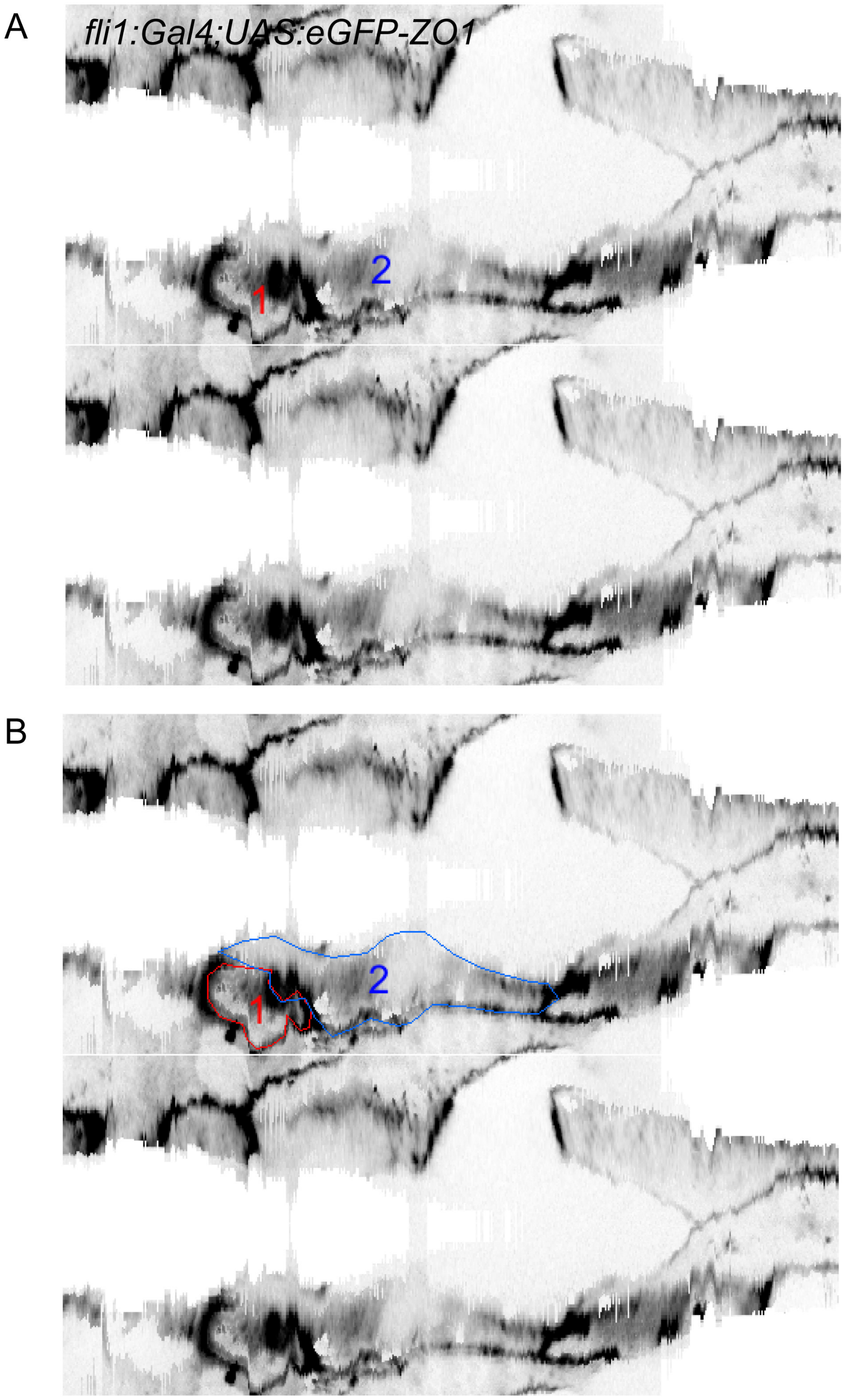

2D projection of DA portion extracted from a confocal TL sequence performed on a 48 hpf *Tg(fli1:Gal4;UAS:RFP; UAS:eGFP-ZO1)* embryo (related to Figure 4-D). (A) Cell numbered in red and blue correspond to “EHT” and “Potentially hemogenic” cells respectively. (B) Same image with the “EHT” and “Potentially hemogenic” cells contours delimited by hand.

Map 3

A

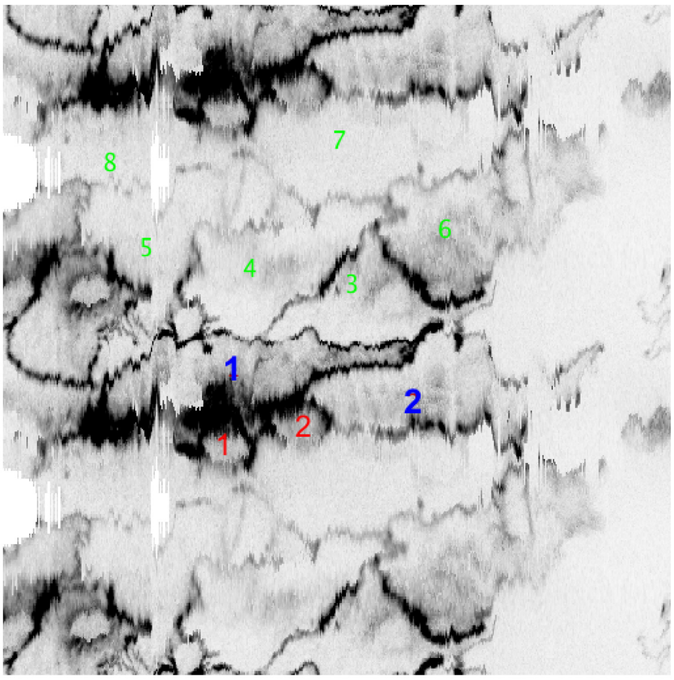

B

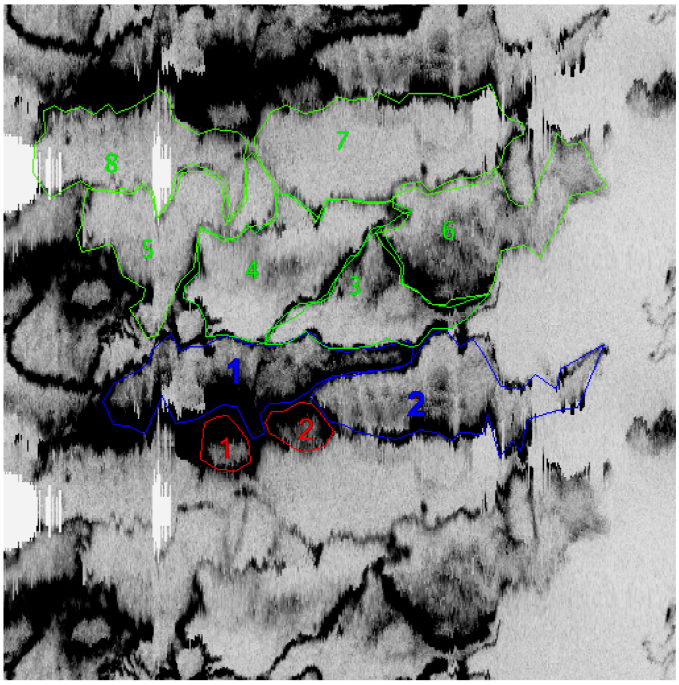

2D projection of DA portion extracted from a confocal TL sequence performed on a 48 hpf *Tg(fli1:Gal4;UAS:RFP; UAS:eGFP-ZO1)* embryo (related to Figure 4-D). (A) Cells numbered in red, blue and green correspond to “EHT”, “Potentially hemogenic” and “Endothelial” cells respectively. (B) Same image with the “EHT”, “Potentially hemogenic” and “EHT” cells contours delimited by hand.

Map 4

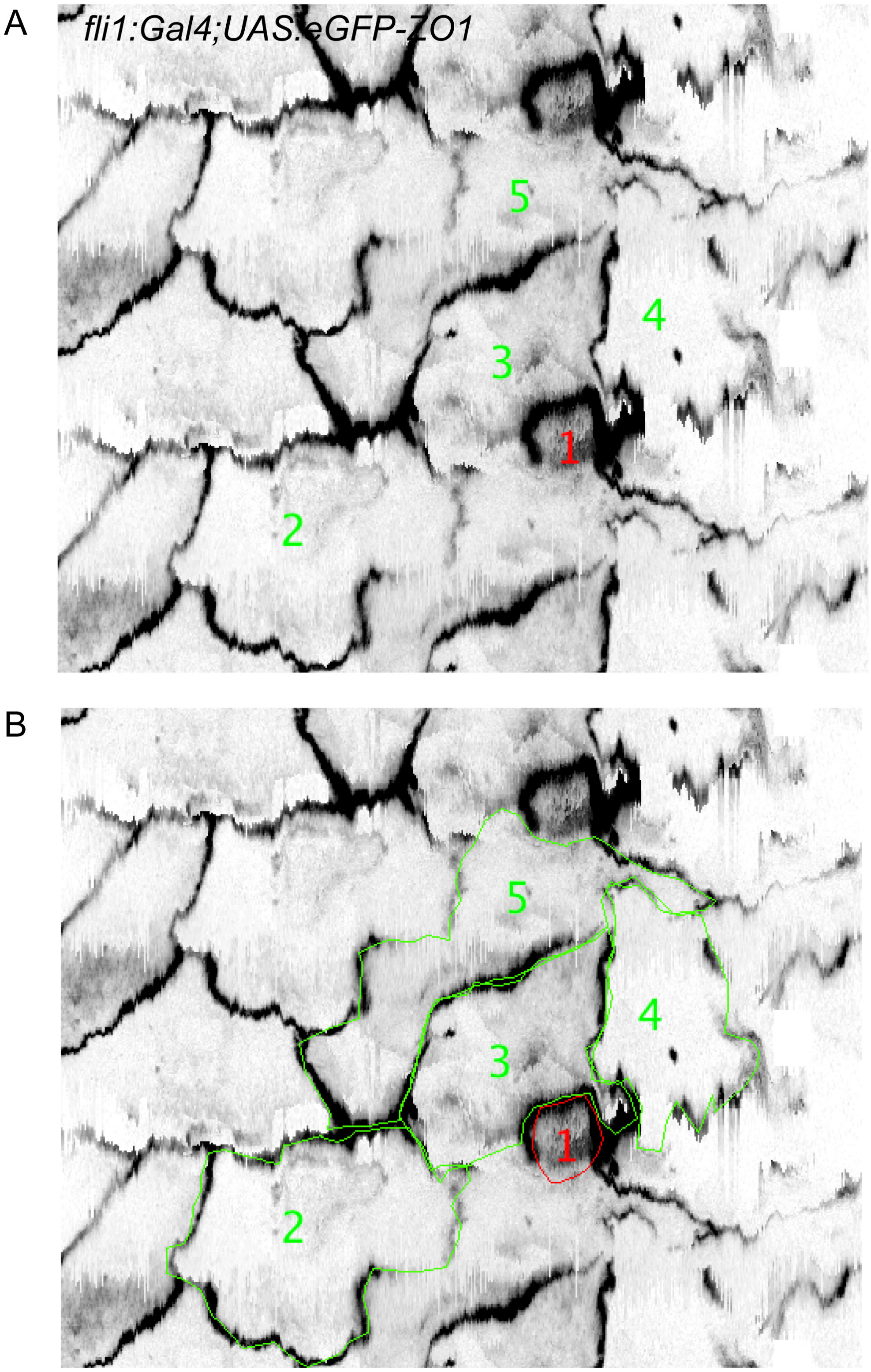

2D projection of DA portion extracted from a single confocal image performed on a 48 hpf *Tg(fli1:Gal4;UAS:RFP; UAS:eGFP-ZO1)* embryo (related to Figure 4-D). (A) Cell numbered in red and green correspond to “EHT” and “Endothelial” cells respectively. (B) Same image with the “EHT” and “EHT” cells contours delimited by hand.

Map 5

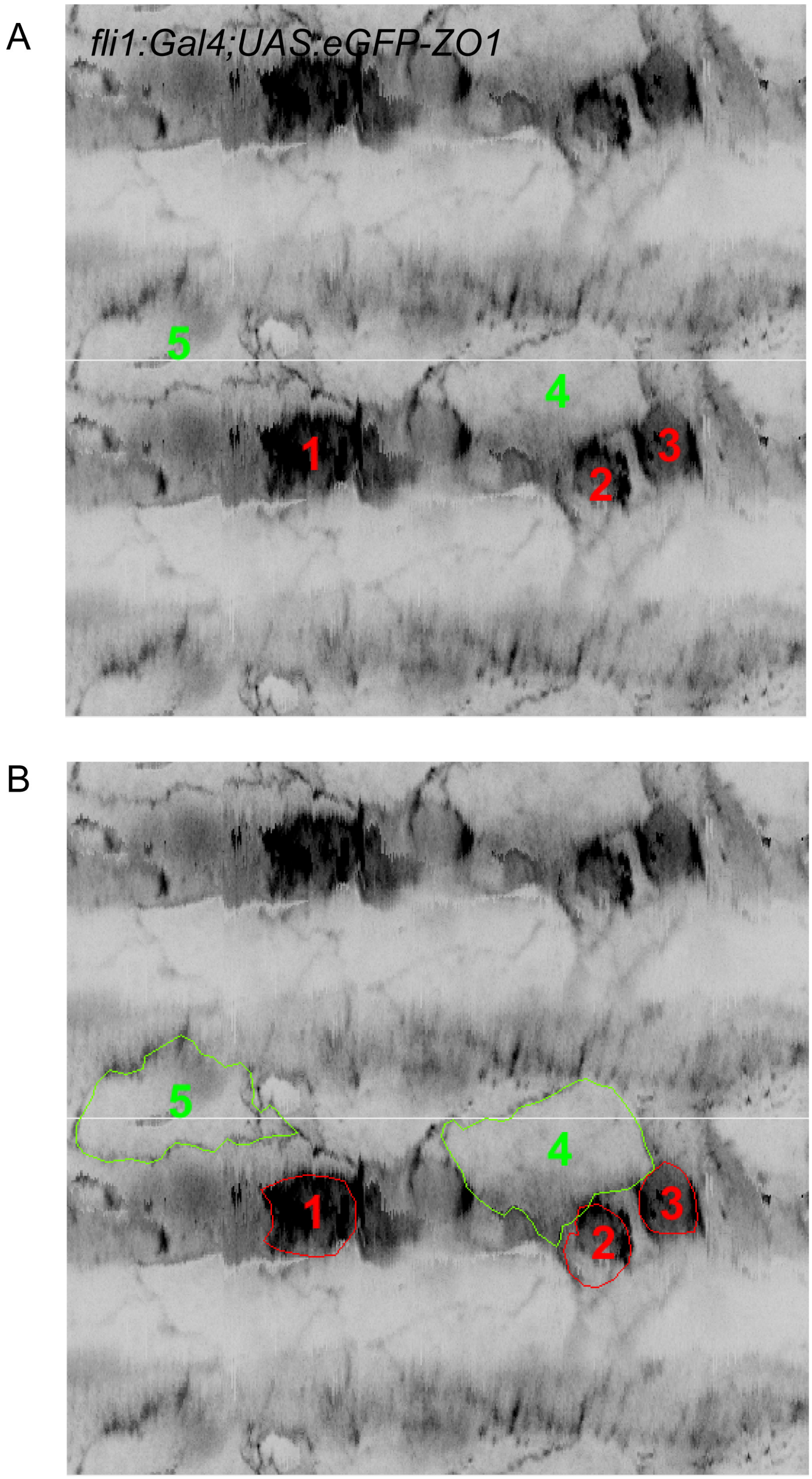

2D projection of DA portion extracted from a confocal TL sequence performed on a 48 hpf *Tg(fli1:Gal4;UAS:RFP; UAS:eGFP-ZO1)* embryo (related to Figure 4-D). (A) Cells numbered in red and green correspond to “EHT” and “Endothelial” cells respectively. (B) Same image with the “EHT” and “EHT” cells contours delimited by hand.

Map 6

A

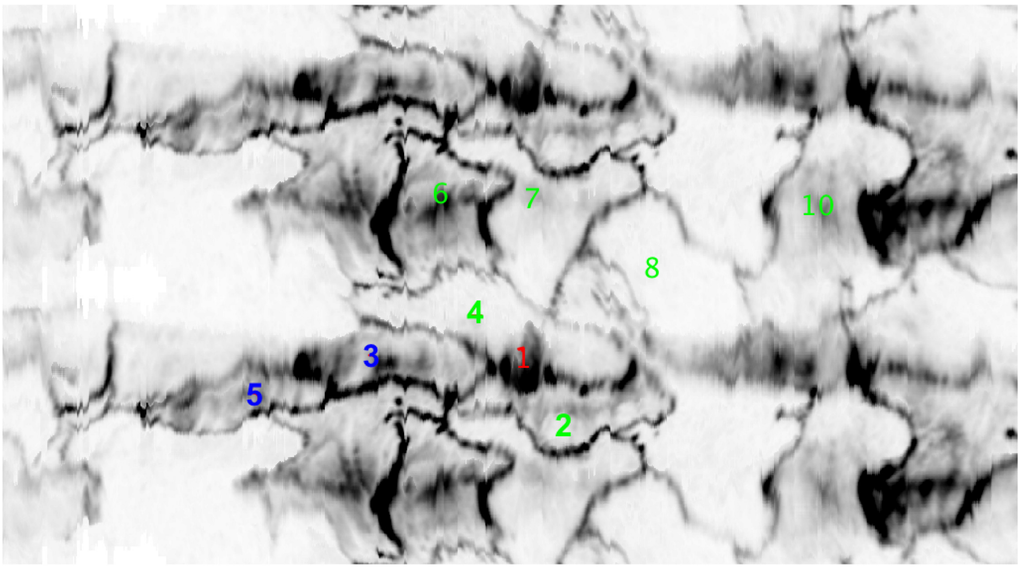

B

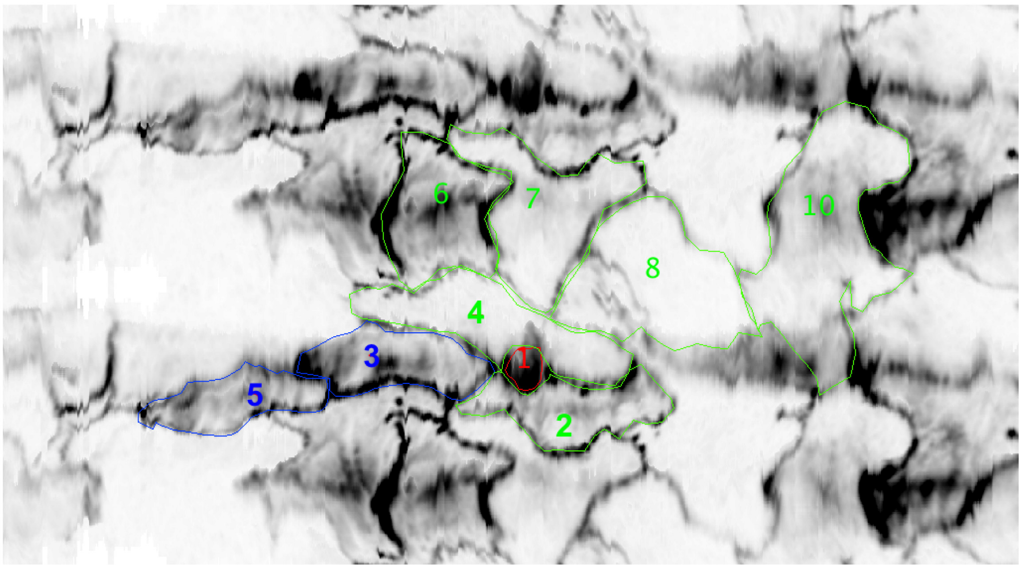

2D projection of DA portion extracted from a confocal TL sequence performed on a 48 hpf *Tg(fli1:Gal4;UAS:RFP; UAS:eGFP-ZO1)* embryo (related to Figure 4-D). (A) Cells numbered in red, blue and green correspond to “EHT”, “Potentially hemogenic” and “Endothelial” cells respectively. (B) Same image with the “EHT”, “Potentially hemogenic” and “EHT” cells contours delimited by hand.
