# Supplementary material for: Anisotropic organization of circumferential actomyosin characterizes hematopoietic stem cells emergence in the zebrafish
Source: eLife. 2018 Aug 22;7:e37355. doi: 10.7554/eLife.37355 (PMC6105311; doi:10.7554/eLife.37355)
Supplement: Figure 5—source data 1. [file elife-37355-fig5-data1.pdf]

Figure 5-source data 1

Figure 5A: Z-projections (on top) and full 2D-Maps (bottom) from which the images presented in the bottom panels of Figure 5A were extracted.

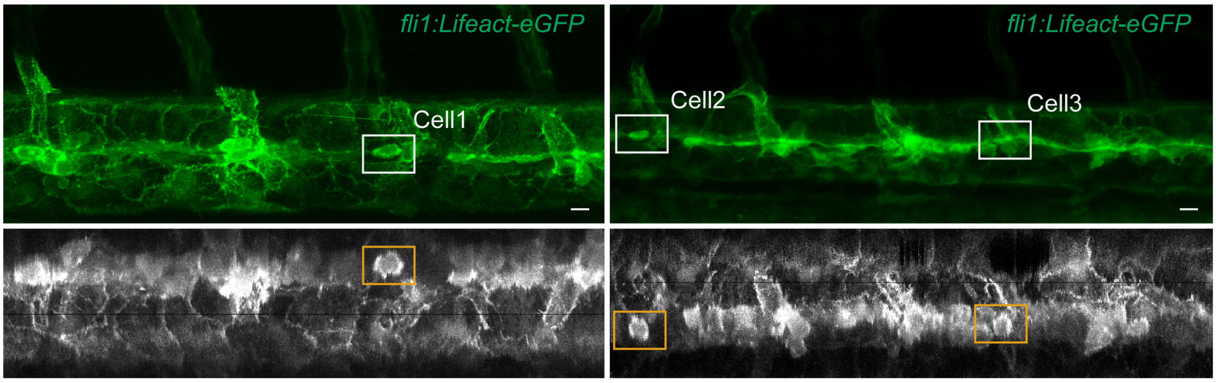

Figure 5-source data 1: Top panels; maximum projection of Z-planes extracted from a single confocal image obtained from a 48 hpf *Tg(fli1:Lifeact-eGFP)* embryo. Bottom panels have been obtained after duplication (x2) of the corresponding 2D-maps (see Methods). White (top) and yellow (bottom) rectangles point at Cells 1, 2 and 3 shown in Figure 5A (bottom panels). Scale bars, 10μm.
