# Supplementary material for: Anisotropic organization of circumferential actomyosin characterizes hematopoietic stem cells emergence in the zebrafish
Source: eLife. 2018 Aug 22;7:e37355. doi: 10.7554/eLife.37355 (PMC6105311; doi:10.7554/eLife.37355)
Supplement: Source code 1. [file elife-37355-code1.docx]

**Source code 1**

Matlab code for tracking, A_P distances through time and closing speeds.

% version 180125 v1.2

function openAndDisplayTrackMateFiles()

clear

close all

filePathSpotFeat = uipickfiles('num',1 ,'Prompt', 'Please select the path to the whole dataset');

% filePathSpotFeat = ...

% {'/media/sherbert/Data/Projects/OG_projects/Project4_ML/movies/160328_projected/280316_extremities1and2.xml'};

[path,fileName,~] = fileparts(filePathSpotFeat{1});

% Use a smoothing factor in the display => Always keep 1 as the first value

% to keep the raw data

smoothFact = [1 5 11]; % to smooth the openings display and analyses

smoothFactAdv = 11; % to apply and advanced smoothing => Change to the number of steps to smooth onto

% Import the data table associated

[ spot_table, spot_ID_map ] = trackmateSpots( filePathSpotFeat{1} );

edge_map = trackmateEdges( filePathSpotFeat{1} );

track_names = edge_map.keys;

n_tracks = numel(track_names);

if n_tracks>2

fprintf('WARNING: %d trajectories detected. Stopping the analysis.\n',...

n_tracks);

return

end

% Double check some parameters of the acquisition

prompt = {'Enter frame interval:', 'Enter time unit:', 'Enter space unit:'};

dlg_title = 'Check inputs';

num_lines = 1;

defaultans = {'2','min','micron'};

answer = inputdlg(prompt,dlg_title,num_lines,defaultans);

md.frameInterval = str2double(answer{1});

md.timeUnits = answer{2};

md.spaceUnits = answer{3};

% Recreate the tracks by spot ID

track_spot_IDs = recreate_IDs(n_tracks, track_names, edge_map);

% Reshape tracks into simpleTracks for simple handling

clipZ = true;

simpleTracks = reshapeTracks(n_tracks, track_spot_IDs,...

spot_ID_map, spot_table, clipZ, md);

% Reshape simpleTracks to incorporate the empty positions => and make it

% Real Time ?

simpleTracksRT = rtTracks(n_tracks, simpleTracks, md);

% Apply smoothing?

%% %%%%%%%%%%%%%%%% 2D only %%%%%%%%%%%%%%%%

% Calculate distance between the 2 extremities

openingRT = InterExtremDist(simpleTracksRT,smoothFact);

% Calculate the opening change

openingRTspeed = [-diff(openingRT) ; NaN(1,numel(smoothFact))]/md.frameInterval;

% Calculate advanced filtering

openingRTadv = advancedFilter(simpleTracksRT, smoothFactAdv, md);

% => first field = distance ; second field = speed;

% Set timecourse

timeCourse = simpleTracksRT{1}.time;

% Preping the legends

for smoothing = 1: numel(smoothFact)

if smoothFact(smoothing)==1

legs{smoothing} = 'No smooth';

else

legs{smoothing} = sprintf('Smooth over %d tp',smoothFact(smoothing));

end

end

%% tracks and surface size

% Display the distance between the 2 extremities and associate tracks

figure;

subplot(2,2,1); % Display the opening size

dispOpeningDia(openingRT, timeCourse, md, legs);

subplot(2,2,2); % Display the tracks

dispTracks(n_tracks, simpleTracksRT, track_names, md);

subplot(2,2,3);

dispOpeningSpeed(openingRTspeed, timeCourse, md, legs);

subplot(2,2,4);

nbins = 20;

dispOpeningHisto(openingRTspeed, md, legs, nbins);

saveas(gcf,sprintf('%s_morphoAnalysis',...

[path, filesep, fileName]));

%% Display the tracks intensities along time

figure;

subplot(2,2,1); % Maximum intensity

dispIntensities(n_tracks, timeCourse, simpleTracksRT, 'maxInt',...

'Max intensity', track_names, md);

subplot(2,2,2); % Total intensity

dispIntensities(n_tracks, timeCourse, simpleTracksRT, 'totInt',...

'Total intensity', track_names, md);

subplot(2,2,3); % Mean intensity

dispIntensities(n_tracks, timeCourse, simpleTracksRT, 'meanInt',...

'Mean intensity', track_names, md);

subplot(2,2,4); % Median intensity

dispIntensities(n_tracks, timeCourse, simpleTracksRT, 'medianInt',...

'Median intensity', track_names, md);

saveas(gcf,sprintf('%s_fluoAnalysis',...

[path, filesep, fileName]));

%% Display overlayed closing speed and closing distance

figure;

dispOverlayDistVsSpeed(timeCourse, openingRT, openingRTspeed);

% saveas(gcf,sprintf('%s_originalOverlay',...

% [path, filesep, fileName], smoothFactAdv));

%% Display overlayed closing speed and closing distance for advanced filtering

figure;

dispOverlayAdvanced(timeCourse, openingRTadv, smoothFactAdv, md);

tempFig = gcf;

saveas(tempFig,sprintf('%s_overlayAdvFiltering_%ddt',...

[path, filesep, fileName], smoothFactAdv));

% saveas(gcf,sprintf('%s_overlayAdvFiltering_%ddt.png',[path, filesep, fileName], smoothFactAdv));

set(tempFig,'PaperOrientation','landscape');

print(tempFig, '-fillpage', '-dpdf', sprintf('%s_overlayAdvFiltering_%ddt.pdf',...

[path, filesep, fileName], smoothFactAdv));

end

function dispIntensities(n_tracks, timeCourse, simpleTracksRT, fieldToPlot,...

varName, track_names, md)

hold on;

for s = 1 : n_tracks

% track_name = track_names{s};

plot(timeCourse, simpleTracksRT{s}.(fieldToPlot), '.-');

end

title(sprintf('%s against time', varName));

xlabel( ['Time (' md.timeUnits ')'] )

ylabel(varName);

legend(track_names, 'Location', 'eastoutside');

end

function [simpleTracksRT, maxTime] = rtTracks(n_tracks, simpleTracks, md)

% Create and pad with nan the empty positions

simpleTracksRT = simpleTracks;

maxTimeTrack = zeros(n_tracks,1);

for tk = 1 : n_tracks

maxTimeTrack(tk) = max(simpleTracks{tk}.frame);

end

maxTime = max(maxTimeTrack);

% Prepare the empty line to add at the tracks

TableFields = simpleTracks{1}.Properties.VariableNames;

emptyLine = num2cell(NaN(1,length(TableFields)));

for tk = 1 : n_tracks % for each track

emptyFrameTable = table;

frameExist = ismember(0:maxTime,simpleTracks{tk}.frame);

for f = 1:length(frameExist) % for each frame

if ~frameExist(f) % if the frame doesn't exist in the original track

% adapt time and frame

emptyLine{1} = f-1;

emptyLine{2} = emptyLine{1}*md.frameInterval;

emptyFrameTable = [emptyFrameTable ; emptyLine];

end

end

if ~isempty(emptyFrameTable)

% Rename the fields of the emptyFrameTable

emptyFrameTable.Properties.VariableNames = TableFields;

% Merge tables of empty and filled frames

simpleTracksRT{tk} = [simpleTracks{tk};emptyFrameTable];

end

% Resort the table based on frames

simpleTracksRT{tk} = sortrows(simpleTracksRT{tk} ,{'frame'},{'ascend'});

end

end

function simpleTracks = reshapeTracks(n_tracks, track_spot_IDs,...

spot_ID_map, spot_table, clipZ, md)

% Reshape the tracks into a table to handle them more easily

simpleTracks = {};

for s = 1 : n_tracks

track_spot_ID = track_spot_IDs{ s };

rows = cell2mat(spot_ID_map.values(num2cell(track_spot_ID)));

frame = spot_table.FRAME(rows);

time = spot_table.FRAME(rows) .* md.frameInterval;

xPos = spot_table.POSITION_X(rows);

yPos = spot_table.POSITION_Y(rows);

if ~clipZ

zPos = spot_table.POSITION_Z(rows);

end

maxInt = spot_table.MAX_INTENSITY(rows);

totInt = spot_table.TOTAL_INTENSITY(rows);

meanInt = spot_table.MEAN_INTENSITY(rows);

medianInt = spot_table.MEDIAN_INTENSITY(rows);

% Could also import the edge displacement but the 'row' order is not very

% clear to me...

% merge data into a table and sort the tracks based on the .frame info

if clipZ

tempTable = table(frame,time,xPos,yPos,maxInt,totInt,meanInt,medianInt);

else

tempTable = table(frame,time,xPos,yPos,xPos,maxInt,totInt,meanInt,medianInt);

end

tempTable = sortrows(tempTable,{'frame'},{'ascend'});

simpleTracks{s} = tempTable;

end

end

function dispTracks(n_tracks, simpleTracksRT, track_names, md)

% Display the tracks of the 2 extremities (2D only for the moment)

hold on

for s = 1 : n_tracks

track_name = track_names{ s};

x = simpleTracksRT{s}.xPos;

y = simpleTracksRT{s}.yPos;

% Plot the tracks by coloring spots.

plot( x, y, '.-' , 'DisplayName', track_name)

end

title('Trajectories of the extremities');

ylabel( [ 'Y (' md.spaceUnits ')' ] )

xlabel( [ 'X (' md.spaceUnits ')' ] )

axis equal

legend toggle

end

function track_spot_IDs = recreate_IDs(n_tracks, track_names, edge_map)

track_spot_IDs = cell( n_tracks, 1 );

% Recreate the tracks by spot ID

for s = 1 : n_tracks

track_name = track_names{s};

edge_table = edge_map( track_name );

track_spot_IDs{ s } = unique( [...

edge_table.SPOT_SOURCE_ID...

edge_table.SPOT_TARGET_ID] );

end

end

function openingRTadv = advancedFilter(simpleTracksRT, smoothFactAdv, md)

% Calculate distance with every filters

diam = InterExtremDist(simpleTracksRT,[1 smoothFactAdv]); % => no smooth and single smooth

diam(:,3) = smooth(diam(:,2),smoothFactAdv); % => 2 smoothing steps

diam = diam .* ~isnan(diam(:,1));

diam(diam==0) = nan;

% Calculate speed with every filters

speed = ([-diff(diam) ; NaN(1,size(diam,2))]/md.frameInterval);

% merge into main structure

openingRTadv.distance = diam;

openingRTadv.speed = speed;

% Prepape legends

openingRTadv.dLegend = {'no smooth diameter',...

sprintf('diameter smoothed once (%ddt)',smoothFactAdv-1),...

sprintf('diameter smoothed twice (%ddt)',smoothFactAdv-1)};

openingRTadv.sLegend = {'no smooth speed',...

sprintf('speed smoothed once (%ddt)',smoothFactAdv-1),...

sprintf('speed smoothed twice (%ddt)',smoothFactAdv-1)};

end

function opening = InterExtremDist(simpleTracksRT,smoothFact)

% show the distance between the 2 extremeties of the cell surface

% 2D only

opening = zeros(length(simpleTracksRT{1}.frame),length(smoothFact));

track1 = [simpleTracksRT{1}.xPos simpleTracksRT{1}.yPos];

track2 = [simpleTracksRT{2}.xPos simpleTracksRT{2}.yPos];

nanTrack = ~isnan(simpleTracksRT{1}.xPos .* simpleTracksRT{2}.xPos);

for smoothing = 1:numel(smoothFact)

opening(:,smoothing) = smooth(sqrt(sum((track2-track1).^2,2)),smoothFact(smoothing));

end

opening = opening.*nanTrack;

opening(opening==0) = nan;

end

function dispOpeningDia(openingRT, timeCourse, md, legs)

% Display the size of the surface diameter (between the extremities)

plot(timeCourse, openingRT, '.-');

title('Distance between extremities');

xlabel( ['Time (' md.timeUnits ')'] );

ylabel( ['Distance (' md.spaceUnits ')' ]);

legend(legs)

end

function dispOpeningSpeed(openingRTspeed, timeCourse, md, legs)

% Display the speed at which the surface is closing

hold on

plot(timeCourse, openingRTspeed, '.-');

title('Closing speed');

xlabel( sprintf('Time (%s)', md.timeUnits) );

ylabel( sprintf('Closing speed (%s/%s)', md.spaceUnits, md.timeUnits) );

legend(legs)

end

function dispOpeningHisto(openingRTspeed, md, legs, nbins)

% Display of the opening speeds as an histogram

lineColors = lines(numel(legs));

minmax = min(min(openingRTspeed)) : ...

abs(min(min(openingRTspeed))/max(max(openingRTspeed)))/nbins : ...

max(max(openingRTspeed));

h = zeros(numel(minmax)-1,numel(legs));

for smoothing = 1:numel(legs)

h(:,smoothing) = histcounts(openingRTspeed(:,smoothing),minmax);

end

histoData = bar(minmax(1:end-1),h);

for pop = 1:numel(legs)

histoData(pop).FaceColor = lineColors(pop,:);

histoData(pop).EdgeColor = 'None';

end

title('Distribution of the closing speeds');

xlabel( sprintf('Closing speed (%s/%s)', md.spaceUnits, md.timeUnits) );

ylabel('N');

legend(legs);

legend boxoff;

end

function dispOverlayDistVsSpeed(timeCourse, openingRT, openingRTspeed)

% Display overlayed closing speed and closing distance

yyaxis left

plot(timeCourse,openingRT);

yyaxis right

plot(timeCourse,openingRTspeed);

end

function dispOverlayAdvanced(timeCourse, openingRTadv, smoothFactAdv, md)

% Display overlayed closing speed and closing distance for advanced filtering

hold on

yyaxis left

plot(timeCourse,openingRTadv.distance);

ylabel( sprintf('Diameter (%s)', md.spaceUnits) );

yyaxis right

plot(timeCourse,openingRTadv.speed);

ylabel( sprintf('Closing speed (%s/%s)', md.spaceUnits, md.timeUnits) );

title(sprintf('Advanced filtering (twice over %ddt <=> %0.1fmin)',...

smoothFactAdv,smoothFactAdv*md.frameInterval));

xlabel( sprintf('Time (%s)', md.timeUnits) );

legend([openingRTadv.dLegend, openingRTadv.sLegend],'Location','EastOutside')

end
