# Supplementary material for: Anisotropic organization of circumferential actomyosin characterizes hematopoietic stem cells emergence in the zebrafish
Source: eLife. 2018 Aug 22;7:e37355. doi: 10.7554/eLife.37355 (PMC6105311; doi:10.7554/eLife.37355)
Supplement: Source code 2. [file elife-37355-code2.docx]

**Source code 2**

Code of the algorithm AortaTracker.

**PART1**

unWrapImage.setData( angle, z, 0, valMax );

                // add point to roi fit

                Point2D point = new Point2D.Double(

                        center.getX() + Math.cos( Math.toRadians( angle ) ) * bestRay,

                        center.getY() + Math.sin( Math.toRadians( angle ) ) * bestRay

                        );

                if ( maxFitROI == null )

                {

                    maxFitROI = new ROI2DPolyLine( point );

                }else

                {

                    maxFitROI.addNewPoint( point , false );

                }

            }

            maxFitROI.setZ( z );

            maxFitROI.setColor( Color.CYAN );

            sourceSequence.addROI( maxFitROI );

            maxFitROI.setName( "tmp fit intensity " + z );

        }

        unWrapImage.endUpdate();

        addSequence( outWrap );

    }

}

**PART2**

package plugins.fab.aorta;

import java.awt.Color;

import java.awt.geom.Point2D;

import icy.image.IcyBufferedImage;

import icy.plugin.abstract_.PluginActionable;

import icy.plugin.interface_.PluginThreaded;

import icy.roi.ROI;

import icy.sequence.Sequence;

import icy.type.DataType;

import plugins.kernel.roi.roi2d.ROI2DEllipse;

import plugins.kernel.roi.roi2d.ROI2DPolyLine;

public class AortaTracker extends PluginActionable implements PluginThreaded {

    @Override

    public void run() {

        System.out.println("Aorta Analysis...");

        // Check sources

        Sequence sourceSequence = getActiveSequence();

        if ( sourceSequence == null )

        {

            System.out.println("A sequence is needed to use this plugin.");

            return;

        }

        // clean of previous ROIs

        for ( int i = sourceSequence.getROIs().size()-1 ; i>=0 ; i-- )

        {

            ROI roi = sourceSequence.getROIs().get( i );

            if ( roi.getName().contains("tmp") )

            {

                sourceSequence.removeROI( roi );

            }

        }

        // tracking.

        System.out.println("Aorta Tracking...");

        ROI2DEllipse roiOut = null;

        try

        {

            roiOut = (ROI2DEllipse) sourceSequence.getROI2Ds().get( 0 );

        }catch( Exception e )

        {

            System.err.println("You need an ROI Ellipse to start.");

            return;

        }

        // create the inner ROI

        int thickness = 15;

        ROI2DEllipse roiIn = new ROI2DEllipse(

                roiOut.getBounds2D().getMinX()+thickness,

                roiOut.getBounds2D().getMinY()+thickness,

                roiOut.getBounds2D().getMaxX()-thickness,

                roiOut.getBounds2D().getMaxY()-thickness );

        roiIn.setName("tmp inner at z=0");

        roiIn.setColor( Color.orange );

        roiIn.setZ( 0 );

        roiOut.setZ( 0 );

        sourceSequence.addROI( roiIn );

        ROI2DEllipse innerPrevious = roiIn;

        ROI2DEllipse outerPrevious = roiOut;

        Sequence outWrap = new Sequence( "outWrap" );

        IcyBufferedImage unWrapImage = new IcyBufferedImage( 360 , sourceSequence.getSizeZ(), 1, DataType.DOUBLE );

        outWrap.addImage( unWrapImage );

        unWrapImage.beginUpdate();

        for ( int z = 1 ; z < sourceSequence.getSizeZ() ; z++ )

        {

            System.out.println("Current Z: " + z );

            // roi to find in current Z

            ROI2DEllipse inner = (ROI2DEllipse) innerPrevious.getCopy();

            ROI2DEllipse outer = (ROI2DEllipse) outerPrevious.getCopy();

            // shift fits.

            IcyBufferedImage image = sourceSequence.getImage( 0 , z );

            short[] data = image.getDataXYAsShort( 0 );

            int window = 5;

            int currentMax = Integer.MIN_VALUE;

            Point2D bestOffset = null;

            for ( int xOffset = -window ; xOffset <= window ; xOffset ++ )

            {

                for ( int yOffset = -window ; yOffset <= window ; yOffset ++ )

                {

                    int val = 0;

                    Point2D center = new Point2D.Double ( outer.getBounds2D().getCenterX() , outer.getBounds().getCenterY() );

                    double rayOuter = outer.getBounds2D().getWidth() /2;

                    double rayInner = inner.getBounds2D().getWidth() /2;

                    for ( float angle = 0 ; angle < 2*3.14d ; angle +=0.1 )

                    {

                        int xx = (int)( xOffset+center.getX() + Math.cos( angle ) * rayOuter );

                        int yy = (int)( yOffset+center.getY() + Math.sin( angle ) * rayOuter );

                        val+= data[yy*image.getWidth()+xx] & 0xFFFF;

                    }

                    for ( float angle = 0 ; angle < 2*3.14d ; angle +=0.1 )

                    {

                        int xx = (int)( xOffset+center.getX() + Math.cos( angle ) * rayInner );

                        int yy = (int)( yOffset+center.getY() + Math.sin( angle ) * rayInner );

                        val-= data[yy*image.getWidth()+xx] & 0xFFFF;

                    }

                    if ( val > currentMax )

                    {

                        currentMax = val;

                        bestOffset = new Point2D.Double( xOffset , yOffset );

                    }

                }

            }

            System.out.println( "Best Offset: " + bestOffset );

            // shift roi.

            inner.translate( bestOffset.getX(), bestOffset.getY() );

            outer.translate( bestOffset.getX(), bestOffset.getY() );

            // display and manage rois.

            inner.setZ( z );

            outer.setZ( z );

            inner.setName( "tmp inner " + z );

            outer.setName( "tmp outer " + z );

            inner.setColor( Color.orange );

            sourceSequence.addROI( inner );

            sourceSequence.addROI( outer );

            innerPrevious = inner;

            outerPrevious = outer;

            // unwrap

            ROI2DPolyLine maxFitROI = null ;

            for ( int angle=0 ; angle < 360 ; angle++ )

            {

                Point2D center = new Point2D.Double ( outer.getBounds2D().getCenterX() , outer.getBounds().getCenterY() );

                double rayOuter = outer.getBounds2D().getWidth() /2;

                // search for a max in a windowRay.

                int windowRay = 15;

                double bestRay = 0;

                double valMax = java.lang.Double.MIN_VALUE;

                for ( double ray = rayOuter - windowRay ; ray < rayOuter + windowRay ; ray++ )

                {

                    int xx = (int)( center.getX() + Math.cos( Math.toRadians( angle ) ) * ray );

                    int yy = (int)( center.getY() + Math.sin( Math.toRadians( angle ) ) * ray );

                    double currentVal = data[yy*image.getWidth()+xx] & 0xFFFF;

                    if ( currentVal > valMax )

                    {

                        valMax = currentVal;

                        bestRay = ray;

                    }

                }
